# Efficacy of sarolaner (Simparica^®^) against induced infestations of *Haemaphysalis longicornis* on dogs

**DOI:** 10.1186/s13071-019-3765-4

**Published:** 2019-10-30

**Authors:** Kenji Oda, Wakako Yonetake, Takeshi Fujii, Andrew Hodge, Robert H. Six, Steven Maeder, Douglas Rugg

**Affiliations:** 1 Research Institute for Animal Science in Biochemistry and Toxicology, 3-7-11, Hashimotodai, Midori-ku, Sagamihara-shi, Kanagawa 252-0132 Japan; 2 Zoetis Japan KK, 3-22-7, Yoyogi, Shibuya-ku, Tokyo 151-0053 Japan; 3 Zoetis Australia Research and Manufacturing, Melbourne, Australia; 4 Zoetis, Veterinary Medicine Research and Development, 333 Portage St., Kalamazoo, MI 49007 USA

**Keywords:** *Haemaphysalis longicornis*, Efficacy, Isoxazoline, Oral, Sarolaner, Simparica^®^, Dog, Tick

## Abstract

**Background:**

*Haemaphysalis longicornis* is the major tick affecting dogs in most of the East Asia/Pacific region and has recently been detected in a number of areas of the USA. This tick is a vector for a number of pathogens of dogs, other mammals and humans. In this study, the efficacy of a single oral administration of sarolaner (Simparica^**®**^, Zoetis) at the minimum label dosage (2 mg/kg) was evaluated against an existing infestation of *H. longicornis* and subsequent weekly reinfestations for 5 weeks after treatment.

**Methods:**

Sixteen dogs were ranked on pretreatment tick counts and randomly allocated to treatment on Day 0 with sarolaner at 2 mg/kg or a placebo. The dogs were infested with *H. longicornis* nymphs on Days − 2, 5, 12, 19, 26 and 33. Efficacy was determined at 48 hours after treatment and subsequent re-infestations based on live tick counts relative to placebo-treated dogs.

**Results:**

There were no adverse reactions to treatment. A single dose of sarolaner provided 100% efficacy on Days 2, 7, 14 and 21; and ≥ 97.4% efficacy on Days 28 and 35. Considering only attached, live ticks, efficacy was 100% for the entire 35 days of the study. Geometric mean live tick counts for sarolaner were significantly lower than those for placebo on all days (11.62 ≤ *t*_(df)_ ≤ 59.99, where 13.0 ≤ *df* ≤ 14.1, *P* < 0.0001).

**Conclusions:**

In this study, a single oral administration of sarolaner at 2 mg/kg provided 100% efficacy against an existing infestation of *H. longicornis* nymphs and ≥ 97.4% efficacy (100% against attached ticks) against weekly reinfestation for at least 35 days after treatment.

## Background

Ticks are a common ectoparasite of dogs globally. As the spectrum and importance of tick-borne diseases in dogs, humans and other animals has expanded, so also has the realization that effective tick control is necessary to help prevent the spread of these diseases, as well as ameliorate the direct clinical impact of tick infestations. *Haemaphysalis longicornis* (Acari: Ixodidae), the Asian longhorned tick bush tick or New Zealand cattle tick, is found in Japan, Korea, eastern China, southeast Russia, Australia, New Zealand, and a number of Pacific islands. Endemic to the East Asia/Pacific region. This three-host tick is thought to have been introduced to Australia in the 19th century on cattle from Japan and from there to New Zealand and other Pacific islands [1]. Cattle are the primary host of *H. longicornis* [2] but adults and nymphs also infest humans and a wide variety of domestic and wild mammals such as dogs, cats, sheep, goats, horses, deer, foxes and rabbits, as well as birds [3, 4]. Larvae are found mainly on small mammals and birds [1]. Extensive infestations can occur causing severe irritation which, combined with blood loss, can lead to weight and productivity losses and even death in cattle, sheep and deer [1, 5, 6]. Through much of its distribution (e.g. New Zealand [7], China [8], Korea [9, 10], and Japan [11]) *H. longicornis* is the most common and/or economically important tick species present. Recently established infestations of *H. longicornis* have been detected in several eastern states in the USA, including the heavily populated suburbs of New York City, and in Arkansas [12, 13].

*Haemaphysalis longicornis* is a potential vector for a wide range of pathogens that affect domestic animals and humans, including bovine theileriosis (*Theileria* spp.), bovine babesiosis (*Babesia ovata*), canine babesiosis (*Babesia gibsoni*), ehrlichiosis (*Ehrlichia* spp.), anaplasmosis (*Anaplasma phagocytophilum*), tick-borne encephalitis (flavivirus), Japanese spotted fever (*Rickettsia japonica*), and an emerging tick-borne zoonosis, severe fever with thrombocytopenia syndrome (phlebovirus) [1, 10, 11, 13–16].

*Haemaphysalis longicornis* is the most prevalent tick found on dogs through most of its Asian distribution. Effective control of this tick is necessary to minimize the adverse clinical effects of infestation as well as to reduce the risk of transmission of the numerous canine and zoonotic pathogens it can vector. Sarolaner is a novel isoxazoline acaricide/insecticide with persistent efficacy following oral administration in dogs [17]. Sarolaner, in a chewable tablet formulation (Simparica^®^, Zoetis) provides control of a fleas and ticks for at least a month after a single oral dose [18] and has been shown to be effective against many tick species globally [19–22].

This study was conducted to evaluate the efficacy of a single administration of sarolaner at the minimum label dose against an existing *H. longicornis* infestation and weekly re-infestations for a period of 5 weeks following treatment.

## Methods

This negative controlled, randomized, laboratory comparative efficacy study was conducted by the Research Institute for Animal Science in Biochemistry and Toxicology (RIAS) at the Narita Animal Science (NAS) Laboratory Co, Ltd, Chiba, Japan. Study procedures followed the World Association for the Advancement of Veterinary Parasitology (WAAVP) guidelines for evaluating the efficacy of parasiticides for the treatment, prevention and control of flea and tick infestation on dogs and cats [23].

### Animals

Eight male and eight female purpose-bred Beagle dogs approximately 22 months of age and weighing 7.8 to 12.1 kg were included in the study. The dogs had not been treated with any acaricidal medications for at least 30 days prior to the study and had not been fitted with acaricidal collars for at least 14 days. Dogs were identified by uniquely numbered ear tattoos and housed individually in indoor enclosures (23 ± 3 °C) that conformed to accepted animal welfare guidelines and ensured no direct contact between dogs. Dogs were acclimatized to these conditions for at least 7 days prior to treatment. Dogs were fed an appropriate ration of a commercial, dry, laboratory-canine feed for the duration of the study. Water was available *ad libitum*. All dogs were given a physical examination to ensure that they were in good health at enrollment and suitable for inclusion in the study. General health observations of each dog were performed at least once daily throughout the study.

### Design

The study followed a randomized complete block design. The 16 dogs were ranked according to pre-treatment tick counts on Day − 5 into blocks of 2, and within each block dogs were randomly allocated to treatment with either placebo or sarolaner, resulting in eight dogs in each treatment group. Dogs were infested with ticks prior to treatment and then re-infested weekly for 5 weeks. Tick counts were conducted 48 h after treatment or re-infestation.

### Treatment

On Day 0, dogs were dosed with either placebo tablets or tablets containing sarolaner. Each dog received from one to two tablets (5 mg, 20 mg) of sarolaner or the equivalent number of placebo tablets to provide the minimum recommended dose of 2 mg/kg (actual range 2.04–2.44 mg/kg) based on body weights taken on Day − 2. Placebo and sarolaner tablet presentations were similar in appearance. Dogs were provided their daily food ration approximately 20 min prior to treatment. All doses were administered by hand pilling to ensure accurate dosing. Each dog was observed for several minutes after dosing for evidence that the dose was swallowed.

### Tick infestation and assessment

Viable, unfed nymphal *H. longicornis* ticks from the NAS Laboratory colony were used for the study. The colony was established from a parthenogenetic strain originally isolated in the Aomori prefecture in May 2002. Nymphs were used for infestation as these attach and feed readily on dogs and are frequently found on dogs in Japan. The preferred hosts for adults are large wild and domestic ruminants [1, 2]. Each dog was infested with 50 *H. longicornis* nymphs at each infestation. Ticks were preferentially placed on the head around the base of the ears to mimic the natural preferred attachment sites. Dogs were sedated with medetomidine hydrochloride (Domitor^®^) to facilitate infestation and were fitted with Elizabethan collars to reduce grooming for the duration of each infestation period, with the exception of the period from Day 0 to Day 2. For tick counts, dogs were sedated and systematically inspected from head to tail, parting the hair by hand, as ticks were observed they were counted and removed. The dogs were then combed thoroughly with a fine-toothed comb. This procedure was conducted for a minimum of 10 min per dog. If ticks were found in the final minute, examination was continued in 1-min increments until no further ticks were found.

Dogs were infested on Day − 7 and live ticks were counted on Day − 5 to ensure the dogs were acceptable hosts and for allocation. The dogs were infested on Day − 2 (2 days before treatment) and assessed on Day 2 to determine efficacy versus an existing infestation. Subsequent weekly infestations were conducted on Days 5, 12, 19, 26 and 33 and counts were performed 48 h after each infestation on Days 7, 14, 21, 28 and 35 to confirm the duration of preventative efficacy. For all post-treatment counts both live and dead ticks were classified as: unattached or free; attached but unengorged; or attached and engorged.

### Statistical analysis

The individual dog was the experimental unit. Tick counts were transformed by the log_e_(count + 1) transformation prior to analysis in order to stabilize the variance and normalize the data. Using the PROC MIXED procedure (SAS 9.2, Cary NC), transformed counts were analyzed using a mixed linear model for repeated measures. The model included the fixed effects of treatment, time point and the interaction between treatment and time point. The random effects included block, animal and error. Two-sided testing was performed at the significance level α = 0.05.

The assessment of efficacy was based on the percent reduction in the arithmetic and geometric mean live tick counts relative to placebo using Abbott’s formula:

$$\% {\text{reduction}} = 100 \times \left[ {{\text{Mean}}\;{\text{count}}\left( {\text{placebo}} \right){-}{\text{Mean}}\;{\text{count}}\left( {\text{treated}} \right)} \right]/{\text{Mean}}\;{\text{count}}\left( {\text{placebo}} \right).$$

## Results and discussion

All dogs included in the study were demonstrated to be acceptable hosts on Day − 5 with an arithmetic mean tick count of 19.5 (range 13–35). The placebo-treated dogs maintained *H. longicornis* infestations throughout the study with two to 32 live ticks recovered from all eight dogs at each examination (Table 1). On Days 2 to 14, all of these ticks were attached. On Days 21 to 35, a small number (1–3) of unattached ticks were found on up to four dogs per time point. The relatively low tick counts for placebo dogs on Day 2 were likely due to the longer infestation period prior to counting (4 days *vs* 2 days for all other counts), removal of Elizabethan collars from Day 0 to Day 2 allowing for greater loss of ticks due to self-grooming by the dogs, and also natural attrition.

**Table 1:** Geometric (arithmetic) mean live *H. longicornis* counts and ranges for dogs treated once orally (Day 0) with placebo or sarolaner chewable tables at 2 mg/kg and percent efficacy relative to placebo at 48 h after treatment and subsequent weekly re-infestations

Day	Placebo			Sarolaner			% Efficacy
	Mean	Range	Dogs with ticks	Mean	Range	Dogs with ticks	
2	4.4 (4.6)	2–7	8/8	0.0^a^ (0.0)	0–0	0/8	100 (100)
7	15.5 (15.6)	13–21	8/8	0.0^a^ (0.0)	0–0	0/8	100 (100)
14	18.3 (18.5)	14–23	8/8	0.0^a^ (0.0)	0–0	0/8	100 (100)
21	18.8 (19.9)	13–32	8/8	0.0^a^ (0.0)	0–0	0/8	100 (100)
28	20.7 (21.1)	13–24	8/8	0.2^a^ (0.3)	0–1	2/7	98.9 (98.7)
35	23.9 (24.1)	20–30	8/8	0.6^a^ (1.0)	0–4	3/7	97.4 (95.9)

^a^Geometric mean live tick count significantly lower than placebo (11.62 ≤ *t*_(df)_ ≤ 59.99, where 13.0 ≤ *df* ≤ 14.1, *P* < 0.0001)

No live ticks were found on any sarolaner-treated dog from Day 2 to Day 21. On Day 28, single live, unattached ticks were recovered from two dogs and on Day 35, three dogs had 1, 2, or 4 live, unattached ticks. Efficacy was 100% from treatment through Day 21 and based on geometric means was 98.9% on Day 28 and 97.4% on Day 35 (Table 1). All of the live ticks recovered from sarolaner-treated dogs were unattached and as these were not feeding they would not have been exposed to sarolaner. Therefore, based on live, attached ticks, efficacy was 100% through the entire study.

There were no adverse reactions to treatment with sarolaner oral tablets. One dog that had been treated with sarolaner (2.23 mg/kg) was found dead on Day 22. This intact male Beagle dog had normal food consumption and was reported to be normal at all general health observations from acclimation until the dog was found dead. A post-mortem examination was conducted on the day after the dog’s death (Day 23). The only finding was congestion of the abdominal and thoracic organs. Detailed review of the necropsy findings and histopathology indicated that the organ congestion was consistent with post-mortem autolysis. The cause of death could not be determined but there was no clear evidence of convulsions. Sarolaner is a gamma-aminobutyric acid antagonist and clinical signs of toxicity are central nervous system excitation. Since, this dog showed no abnormal signs in the 3 weeks between administration of sarolaner up until its death, treatment is considered unlikely to have a causal relationship with its death.

This study evaluated efficacy against the nymphal stage of *H. longicornis* as this stage is commonly found infesting dogs. Similar high level of efficacy could be expected against adult *H. longicornis* as sarolaner has demonstrated month long efficacy against the adults of many tick species globally [19–22]. Another oral isoxazoline, afoxolaner, has been evaluated against adult *H. longicornis* and produced efficacy > 91.9% (based on live tick counts) up to 30 days after a single treatment [24] and recently a third isoxazoline, lotilaner, provided efficacy of > 95% against adults for up to 35 days after a single oral dose [25], while a fourth, fluralaner resulted in efficacy of 93.6% or more for 114 days after the recommended dosage [26]. It should be noted that efficacy in the latter two studies was based on counts of live attached ticks only; when sarolaner was assessed using attached ticks, efficacy was 100% for the entire 35 days. Although the four studies cannot be directly compared due to the differences in design (only assessing live attached ticks) and stage evaluated (nymphs in this study), the general excellent acaricidal efficacy of the isoxazolines was confirmed against *H. longicornis*. The high relative potency of sarolaner compared to the other compounds was demonstrated as efficacy assessed *versus* total live tick counts was always 97.4% or greater through Day 35 and was 100% for the entire study when determined for live attached ticks. Sarolaner has also shown a more rapid speed of kill and greater sustained efficacy than afoxolaner against several tick species [21, 27–29]. A rapid speed of kill is an advantage for an orally administered acaricide as this limits the time that ticks feed before receiving an effective dose, thus reducing the chances of the transmission of tick-borne pathogens. Recently, a single oral treatment with oral sarolaner has been demonstrated to prevent the transmission of *Babesia cani*s to dogs from infected *Dermacentor reticulatus* ticks [30].

Systemic medications for the prevention of tick infestations have advantages over topical formulations, including ease of accurate dosing, no exposure to topical pesticide residues in the hair coat and no reduction in the duration of efficacy due to shampooing, rain or swimming etc. *Haemaphysalis longicornis* is the most common tick infesting dogs in East Asia and is the vector of numerous pathogens of dogs (including babesiosis) as well as zoonoses that cause serious even fatal diseases. The rapid and complete control of *H. longicornis* provided for up to 35 days after a single oral treatment in this study confirms that treatment of dogs with Simparica^®^ would provide effective control of the important ticks afflicting dogs and potentially reduce the risk of transmission of tick-borne pathogens.

## Conclusions

A single oral dose of sarolaner (Simparica^®^) at the minimum recommended dose of 2 mg/kg effectively treated an existing infestation of *H. longicornis* nymphs and prevented re-infestation for up to 35 days. This convenient chewable formulation of sarolaner is a valuable tool for the treatment and prevention of tick infestations and the reduction of the risk of transmission of tick-borne diseases in dogs.

## Data Availability

The dataset supporting the conclusions of this article is included within the article.
